# Structural parameters of dimethyl sulfoxide, DMSO, at 100 K, based on a redetermination by use of high-quality single-crystal X-ray data

**DOI:** 10.1107/S2056989017012464

**Published:** 2017-09-05

**Authors:** Hans Reuter

**Affiliations:** a Institute of Chemistry of New Materials, University of Osnabrück, Barbarastrasse 7, 49069 Osnabrück, Germany

**Keywords:** crystal structure, dimethyl sulfoxide, van der Waals inter­action, geometric parameters, crystal packing

## Abstract

The title compound, C_2_H_6_OS, is a high melting, polar and aprotic solvent widely used in organic and inorganic chemistry. It serves as a H-atom acceptor in hydrogen bonding and is used as an ambidentate ligand in coordination chemistry. The evaluation of the influence of inter­molecular inter­actions on the inter­nal structural parameters of the chemically bonded DMSO mol­ecules affords precise structural data of the free mol­ecule as a point of reference. So far, valid data have been obtained only by use of neutron powder diffraction [Ibberson (2005 ▸). *Acta Cryst.* C**61**, o571–o573]. In the present redetermination, structural data have been obtained from a single-crystal X-ray diffraction experiment at 100 K, revealing a better comparison with DMSO mol­ecules in other crystal structures. In the solid state, the pyramidal mol­ecule exhibits a nearly perfect *C*_s_ symmetry [including H atoms, which are eclipsed with respect to the C⋯C axis], with a C—S—C bond angle of 97.73 (7)° and an S—O bond length of 1.5040 (10) Å, corresponding very well with an S=O double bond, and with almost equal S—C bond lengths [mean value = 1.783 (4) Å] and O—S—C bond angles [mean value = 106.57 (4)°]. The crystal packing is influenced by C—H⋯O inter­actions (2.42–2.47 Å) between all three H atoms of only one methyl group with the O atoms of three neighbouring DMSO mol­ecules. The inter­actions of the O atom with H atoms (or Lewis acids, or hydrogen-donor groups) of adjacent mol­ecules in relation to the orientation of the complete DMSO mol­ecule are described in terms of the angle ω and the distance *d*_norm_; ω is the angle between the pseudo-mirror plane of the mol­ecule and the plane defined through the S=O bond and the inter­acting atom, and *d*_norm_ is the distance of the inter­acting atom from the plane perpendicular to the S=O bond.

## Abstract 2

Accurate structural parameters (bond lengths and angles) of dimethyl sulfoxide, DMSO, have been obtained from the redetermination of its crystal structure by single-crystal X-ray diffraction at 100 K using CCD data in order to get a reference point for the deformation of the chemically bonded mol­ecule. In addition, the new data show that mol­ecule approximates *C*_s_ symmetry in the solid state where all atoms occupy general positions.

## Chemical context   

Dimethyl sulfoxide (DMSO), (CH_3_)_2_SO, is a colourless polar aprotic solvent with high melting (291 K) and boiling points (462 K), miscible with a wide range of organic solvents and water. It is commonly used in organic and inorganic chemistry because of its capability to dissolve numerous polar or nonpolar compounds. In addition to its solvation properties, the mol­ecule may act as a H-atom acceptor in hydrogen bonding, as well as an ambidentate Lewis base in coordination compounds. In the latter case, DMSO reactivity follows the HSAB principle (Pearson, 1963 ▸) which means that in combination with ‘hard’ acids like tin(IV), DMSO coordinates *via* the ‘hard’ O atom [*e.g.* iPrSnCl_3_(DMSO-O)_2_; Kastner & Reuter, 1999 ▸] and in combination with ‘soft’ acids like platinum(II) *via* the ‘soft’ S atom [*e.g. cis*-PtCl_2_(DMSO-S)_2_; Melanson & Rochon, 1975 ▸], while with acids at the ‘hard–soft’ borderline like ruthenium(II), both coordination modes can be realized [*cis*-RuCl_2_(DMSO-O)_1_(DMSO-S)_3_; Tarighi & Abbasi, 2007 ▸]. DMSO is also used in pharmacology in transdermal drug delivery applications and in veterinary medicine.
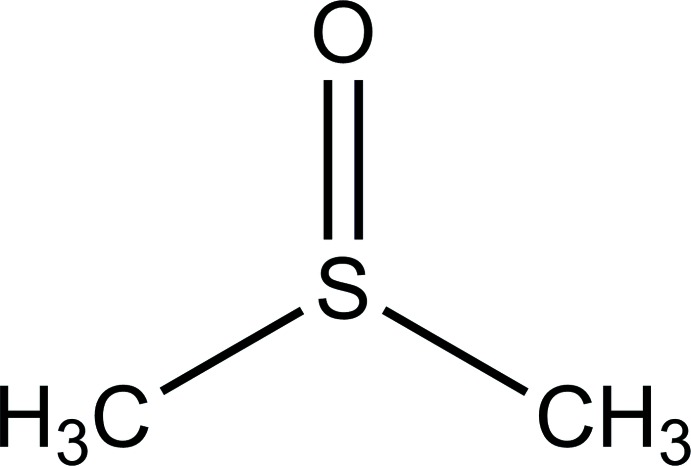


Both hydrogen-bond formation and formation of coordination bonds will change the structural parameters of the DMSO mol­ecule, as was shown by Calligaris (2004 ▸) for DMSO and other sulfoxides. For the evaluation of the influence of these additional inter­molecular bonds on the inter­nal structural parameters of the coordinating or hydrogen-bonded DMSO ligands, precise data on bond lengths and angles within the free mol­ecule are required as a point of reference. The available data, however, in the case of single-crystal X-ray structure determinations, are from the late 1960s (Viswamitra & Kannan, 1966 ▸; Thomas *et al.*, 1966 ▸) when precession and Weissenberg photographs were state of the art. Therefore, these data are of less accuracy compared with modern X-ray data obtained with CCD area detectors. More recently, Ibberson (2005 ▸) published results on neutron powder diffraction studies of fully deuterated dimethyl sulfoxide at 2 and 100 K. Although, the data obtained are of higher precision than those of the forgoing single-crystal X-ray measurements, they suffer from the limitations of powder diffraction techniques.

In the current study, the results of a redetermination of the crystal structure of DMSO based on single-crystal X-ray data at 100 K are presented. The results are comparatively discussed with the previous structure determinations.

## Structural commentary   

**Figure 1:**
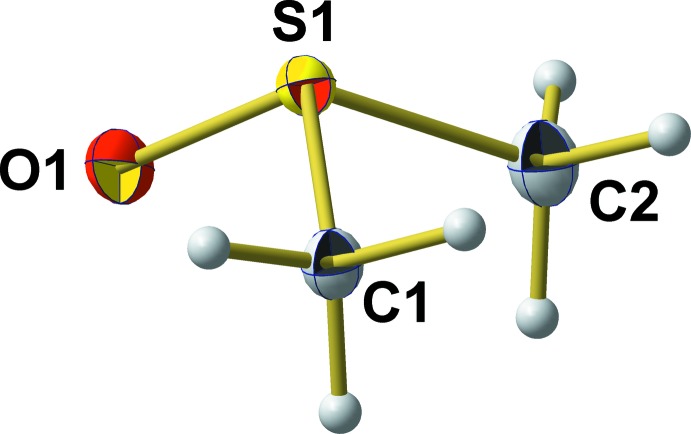
The molecular structure of the title compound, showing the atom-labelling scheme and displacement ellipsoids for the non-H atoms at the 50% probability level.

Unit-cell parameters of the current 100 K single-crystal X-ray measurement (SCXD) are consistent with those of the neutron powder diffraction (NPD) data of Ibberson (2005 ▸), but structural parameters of the DMSO mol­ecule differ considerably between the two refinements (Table 1 ▸). In the pyramidal mol­ecule of crystallographic point group symmetry *C*_1_ (Fig. 1 ▸, atom positions and atom labelling according to NPD), the S atom lies 0.6994 (9) Å above the triangular base formed by the O and C atoms. The S—O bond length of 1.5040 (10) Å is slightly longer than the value [1.496 (2) Å] determined by Ibberson at 100 K, but corresponds very well with a S=O double bond in sulfoxides [1.497 (13) Å; Allen *et al.*, 1987 ▸].

Other differences between the single-crystal X-ray and neutron powder diffraction data, however, are strongly expressed with respect to S—C bond lengths and even more with respect to O—S—C bond angles (Table 1 ▸). In the case of the neutron data, the difference between both S—C bonds is 0.05 Å [S—C1 = 1.838 (3) Å and S—C2 = 1.788 (3) Å], while in the case of the X-ray data, the difference between both bonds is reduced by a factor of about 10 to 0.006 Å [S—C1 = 1.7801 (14) Å and S—C2 = 1.7861 (15) Å]. Moreover, the bond to atom C1 is shorter than the bond to C2, in contrast to the bond-length distribution observed by Ibberson. This is of special inter­est in view of the C—H⋯O inter­actions discussed below. With respect to the C—S—O bond angles, structural differences between the NPD and SCXD model are enormous: the difference between both bond angles of 3.09° [O—S—C1 = 105.21 (16)° and O—S—C2 = 108.30 (15)°] found by Ibberson at 100 K can be compared with a difference of only 0.06° [O—S—C1 = 106.54 (6)° and O—S—C2 = 106.60 (7)°] in the case of the present work. All in all, the ideal *C*_*s*_ point group symmetry of the gaseous and liquid DMSO mol­ecule is much better approached in the crystalline state, even at 100 K, than originally assumed from neutron powder data.

Although this symmetry consideration is not affected by the bond angle between the S atom and the methyl groups, it is important – on the background of coordination that seems to have a great influence on this bond angle – to emphasize that in the SCXD model [C—S—C = 97.73 (3)°], this angle is about 1.4° larger than in the NPD model [C—S—C = 96.37 (12)° at 100 K]. With respect to the hydrogen/deuterium positions, no differences occur, as both methyl groups show an eclipsed orientation with respect to C⋯C, thus fulfilling the nearly ideal *C_s_* symmetry, too.

For the sake of completeness, structural data of the previous single-crystal X-ray structure determination by Thomas *et al.* (1966 ▸) are also compiled in Table 1 ▸.

## Supra­molecular features   

**Figure 2:**
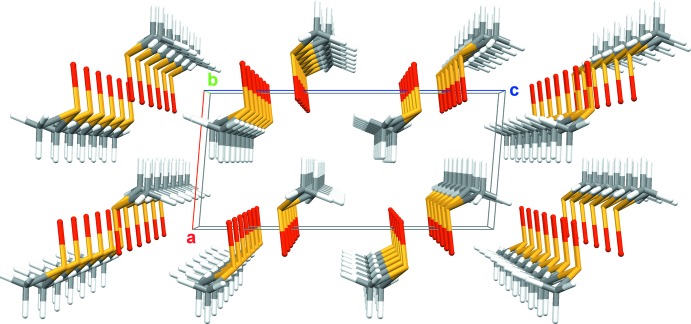
Crystal packing showing the tube-like arrangment of the mol­ecules along the *b* axis.

**Figure 3:**
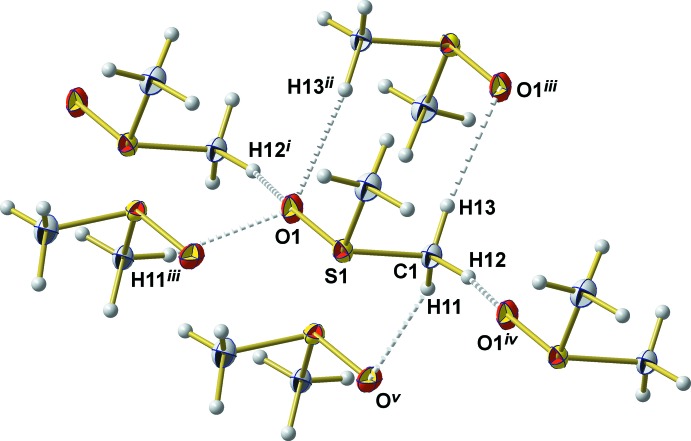
C—H⋯O contacts (grey brocken sticks) between the DMSO mol­ecule and its neighbours. [Symmetry codes used to generate equivalent atoms: (i) 1 − *x*, 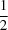 + *y*, 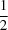 − *z*; (ii) −1 + *x*, *y*, *z*; (iii) −*x*, −*y*, −*z*; (iv) 1 + *x*, *y*, *z*; (v) −*x*, −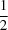 + *y*, 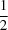 − *z*.]

**Figure 4:**
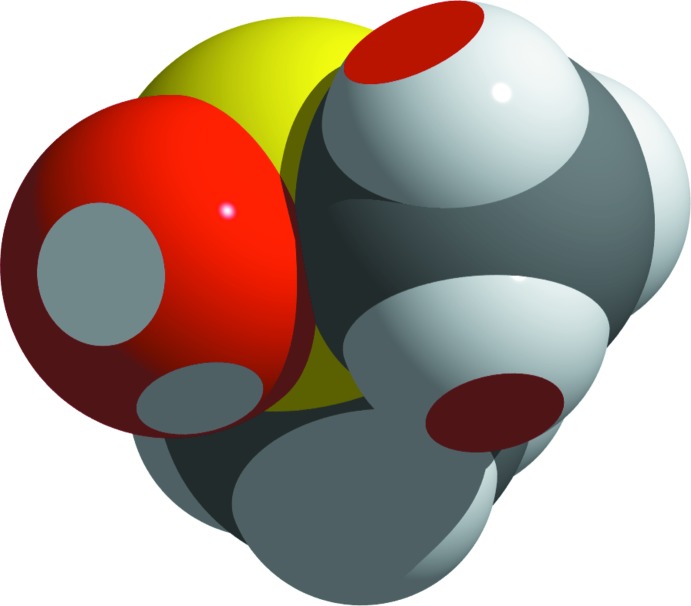
Space-filling model of the DMSO mol­ecule showing four of the six C—H⋯O contacts. Spheres overlap has been visualized by removing the resulting caps.

C—H⋯O contacts are the most prominent inter­molecular inter­actions responsible for the three-dimensional arrangement of the DMSO mol­ecules in the solid state (Fig. 2 ▸). In order to compare our results with the results of the neutron powder diffraction experiment, one must take into account the different validity and refinement strategies for the H/D atoms in both methods. Under consideration of the van der Waals radii of H (1.10 Å) and O (1.52 Å) supplied by Mantina *et al.* (2009 ▸), relevant H⋯O distances should be shorter than 2.62 Å. From the H atoms attached to C2, only one (H22) shows an inter­atomic distance below this threshold. With an H22⋯O1^ii^ (for symmetry code, see Table 2 ▸) distance of 2.61 Å, a binding C—H⋯O inter­action other than a van der Waals inter­action can be excluded. Just the opposite is observed in case of the H atoms attached to C1: all three H atoms show an inter­molecular contact to one O atom of three different DMSO mol­ecules in the range 2.42–2.47 Å (Table 2 ▸). In this case, these contacts fall below the van der Waals distance by 7.6–5.7% (0.20–0.15 Å) which justifies the assumption of binding C—H⋯O inter­actions. The corresponding inter­molecular donor–acceptor distances are in the range 3.318 (2)–3.445 (2) Å, while the C—H⋯O angles are in the range 152.0–173.0° (Table 2 ▸). In summary, each DMSO mol­ecule participates in six C—H⋯O contacts to five neighbouring mol­ecules (Fig. 3 ▸). The extent of the van der Waals and hydrogen-bonding inter­actions on the overlapping of the mol­ecules is visualized in Fig. 4 ▸. Obviously, there is no weakening influence of these inter­actions on the S—C bond length. Quite the opposite, the S1—C1 bond is somewhat shorter than the S1—C2 bond (see above).

**Figure 5:**
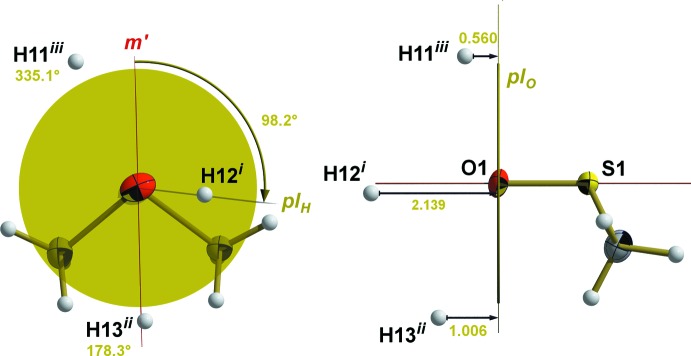
Geometrical boundary conditions for the determination of ω (left) and *d*_norm_ (right, distances in Å) by use of the pseudo-mirror plane *m*′ (definition: O1, S1, mid-point between C1 and C2), the plane *pl_H_* (definition: the inter­acting atom, S=O bond), and the plane *pl_O_* (definition: O1, S=O bond = normal vector). [Symmetry codes used to generate equivalent atoms: (i) 1 − *x*, 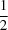 + *y*, 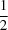 − *z*; (ii) −1 + *x*, *y*, *z*; (iii) −*x*, −*y*, −*z*.]

With respect to the O atom as an acceptor atom, bond angles (S=O⋯H) of the van der Waals contacts come to 114.0° for H13^2^ (^2^ = −1 + *x*, *y*, *z*), 152.1° for H12^1^ (^1^ = −*x*, 0.5 + *y*, 0.5 − *z*), and 103.4° for H11^3^ (^3^ = −*x*, −*y*, −*z*). The geometrical aspects of these van der Waals inter­actions (or of coordinatively or hydrogen-bonded DMSO mol­ecules) are described only incompletely with the foregoing used distances and angles as they disregard the orientation of the complete DMSO mol­ecule in relation to the inter­actions described. In order to unambiguously account for this specific relationship, indexation by two additional values, ω and *d*_norm_, using two planes as a reference (Fig. 5 ▸) is suggested. The first plane is identical, with the pseudo-mirror plane *m*′ defined by O1, S1 and the mid-point between both C atoms. The second plane, *pl_O_*, is perpendicular to the S=O bond and located in O1. While *d*_norm_ represents the distance between the inter­acting atom (*via* a van der Waals inter­action, a hydrogen bond or a coordinative bond) and *pl_O_*, the angle ω marks the angle between *m*′ and the plane *pl_H_* defined by O1, S1 and the inter­acting atom. Values of ω can stretch from 0 to 360° when looking down the O=S bond as in a Newman projection. In the case of the van der Waals inter­actions discussed here, the corresponding ω/*d*_norm_ values are: H12^1^ = 98.2°/2.139 Å, H13^2^ = 178.3°/1.006 Å and H11^3^ = 335.1°/0.560 Å.

## Synthesis and crystallization   

Single crystals were grown from a commercial available sample (Sigma–Aldrich) within a 0.3 mm thick Lindemann capillary using the Kryoflex low-temperature device of the diffractometer.

## Refinement   

Crystal data, data collection and structure refinement details are summarized in Table 3 ▸. All six H atoms were found in a difference-Fourier map. They could be refined without any restraints in meaningful positions [C—H range = 0.91 (2)–0.97 (2) Å; H—C—H range = 107.6 (11)–112.2 (15)°] with individual isotropic displacement parameters [range = 0.018 (4)–0.036 (5) Å^2^]. In order to obtain a structure model comparable to typical refinement techniques of DMSO mol­ecules in the structures of coordination compounds or with hydrogen bonds, conventional constraints [AFIX 137, C—H = 0.99 Å, H—C—H = 109.6° in *SHELXL* (Sheldrick, 2015 ▸)] with two common isotropic displacement parameters, one for each methyl group, have been applied. In summary, these restraints only slightly affected the final results: the final *R* value increased from 2.37 to 2.42%, while the bond lengths and bond angles remained unchanged. All data have been approved by a second independently grown crystal.

## Supplementary Material

Crystal structure: contains datablock(s) I. DOI: 10.1107/S2056989017012464/wm5409sup1.cif

Structure factors: contains datablock(s) I. DOI: 10.1107/S2056989017012464/wm5409Isup2.hkl

Supporting information file. DOI: 10.1107/S2056989017012464/wm5409Isup3.cml

Supporting information file. DOI: 10.1107/S2056989017012464/wm5409sup3.mp4

CCDC reference: 1571260

Additional supporting information:  crystallographic information; 3D view; checkCIF report

## Figures and Tables

**Table 1:** Experimental details of previous crystal structure determinations of DMSO and their comparison with the present study

	Thomas *et al.* (1966 ▸)	Ibberson (2005 ▸)	This work
Space group, *Z*	*P*2_1_/*c*, 4	*P*2_1_/*c*, 4	*P*2_1_/*c*, 4
*a* (Å)	5.303 (5)	5.2390 (1)	5.2243 (3)
*b* (Å)	6.829 (3)	6.7581 (1)	6.7414 (4)
*c* (Å)	11.693 (3)	11.2696 (1)	11.2772 (6)
*β* (°)	94.5 (3)	94.8053 (3)	94.820 (2)
*V* (Å^3^)	422.2	397.60 (1)	395.77 (4)
*T* (K)	278	100	100
Sample	single-crystal	powder	single-crystal
Radiation	Mo *K*α	neutron	Mo *K*α
Technique	precession photographs	HRPD	CDC
*R* value	7.4%	3.77%	2.4%
Number of reflections	777	not given	938
Number of parameters	not given	93	41
H(D) atoms	constrained	refined	constrained
*d*(S1—O1) (Å)	1.531 (5)	1.496 (2)	1.5040 (10)
*d*(S1—C1) (Å)	1.775 (8)	1.838 (3)	1.7801 (14)
*d*(S1—C2) (Å)	1.821 (11)	1.788 (3)	1.7861 (15)
O1—S1—C1 (°)	106.7 (4)	105.2 (2)	106.54 (6)
O1—S1—C2 (°)	106.8 (4)	108.3 (2)	106.60 (7)
C1—S1—C2 (°)	97.4 (4)	96.4 (1)	97.73 (7)

**Table 2:** Hydrogen-bond geometry (Å, °)

*D*—H⋯*A*	*D*—H	H⋯*A*	*D*⋯*A*	*D*—H⋯*A*
C1—H11⋯O1^i^	0.98	2.42	3.3318 (18)	155
C1—H12⋯O1^ii^	0.98	2.42	3.3184 (17)	152
C1—H13⋯O1^iii^	0.98	2.47	3.4450 (19)	173
C2—H22⋯O1^ii^	0.98	2.61	3.4618 (18)	146

Symmetry codes: (i) 
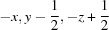
; (ii) 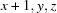; (iii) 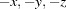.

**Table 3:** Experimental details

Crystal data	
Chemical formula	C_2_H_6_OS
*M*_r_	78.13
Crystal system, space group	Monoclinic, *P*2_1_/*c*
Temperature (K)	100
*a*, *b*, *c* (Å)	5.2243 (3), 6.7414 (4), 11.2772 (6)
β (°)	94.820 (2)
*V* (Å^3^)	395.77 (4)
*Z*	4
Radiation type	Mo *K*α
μ (mm^−1^)	0.60
Crystal size (mm)	0.21 × 0.17 × 0.16

Data collection	
Diffractometer	Bruker APEXII CCD
Absorption correction	Multi-scan (*SADABS*; Bruker, 2009 ▸)
*T*_min_, *T*_max_	0.883, 0.913
No. of measured, independent and observed [*I* > 2σ(*I*)] reflections	5476, 938, 801
*R*_int_	0.030
(sin θ/λ)_max_ (Å^−1^)	0.659

Refinement	
*R*[*F* ^2^ > 2σ(*F* ^2^)], *wR*(*F* ^2^), *S*	0.024, 0.062, 1.13
No. of reflections	938
No. of parameters	41
H-atom treatment	H-atom parameters constrained
Δρ_max_, Δρ_min_ (e Å^−3^)	0.26, −0.25

Computer programs: *APEX2* (Bruker, 2009 ▸), *SAINT* (Bruker, 2009 ▸), *SHELXS97* (Sheldrick, 2008 ▸), *SHELXL2014* (Sheldrick, 2015 ▸), *DIAMOND* (Brandenburg, 2006 ▸), *Mercury* (Macrae *et al.* (2008 ▸) and *SHELXTL* (Sheldrick, 2008 ▸).

## supplementary crystallographic information

### Crystal data

**Table:**

C_2_H_6_OS	*F*(000) = 168
*M**_r_* = 78.13	*D*_x_ = 1.311 Mg m^−^^3^
Monoclinic, *P*2_1_/*c*	Mo *K*α radiation, λ = 0.71073 Å
*a* = 5.2243 (3) Å	Cell parameters from 3349 reflections
*b* = 6.7414 (4) Å	θ = 3.5–28.0°
*c* = 11.2772 (6) Å	µ = 0.60 mm^−^^1^
β = 94.820 (2)°	*T* = 100 K
*V* = 395.77 (4) Å^3^	Bloc, colourless
*Z* = 4	0.21 × 0.17 × 0.16 mm

### Data collection

**Table:**

Bruker APEXII CCD diffractometer	801 reflections with *I* > 2σ(*I*)
φ and ω scans	*R*_int_ = 0.030
Absorption correction: multi-scan (SADABS; Bruker, 2009)	θ_max_ = 28.0°, θ_min_ = 3.5°
*T*_min_ = 0.883, *T*_max_ = 0.913	*h* = −6→6
5476 measured reflections	*k* = −8→8
938 independent reflections	*l* = −14→14

### Refinement

**Table:**

Refinement on *F*^2^	0 restraints
Least-squares matrix: full	Hydrogen site location: inferred from neighbouring sites
*R*[*F*^2^ > 2σ(*F*^2^)] = 0.024	H-atom parameters constrained
*wR*(*F*^2^) = 0.062	*w* = 1/[σ^2^(*F*_o_^2^) + (0.0238*P*)^2^ + 0.1531*P*] where *P* = (*F*_o_^2^ + 2*F*_c_^2^)/3
*S* = 1.13	(Δ/σ)_max_ < 0.001
938 reflections	Δρ_max_ = 0.26 e Å^−^^3^
41 parameters	Δρ_min_ = −0.25 e Å^−^^3^

### Special details

**Table:**

Geometry. All esds (except the esd in the dihedral angle between two l.s. planes) are estimated using the full covariance matrix. The cell esds are taken into account individually in the estimation of esds in distances, angles and torsion angles; correlations between esds in cell parameters are only used when they are defined by crystal symmetry. An approximate (isotropic) treatment of cell esds is used for estimating esds involving l.s. planes.

### Fractional atomic coordinates and isotropic or equivalent isotropic displacement parameters (Å^2^)

**Table:**

	*x*	*y*	*z*	*U*_iso_*/*U*_eq_	
S1	0.18232 (6)	0.14961 (5)	0.19283 (3)	0.01776 (12)	
O1	−0.10440 (18)	0.13580 (16)	0.16758 (10)	0.0245 (3)	
C1	0.3139 (3)	−0.0633 (2)	0.12708 (13)	0.0187 (3)	
H11	0.2663	−0.1825	0.1698	0.027 (3)*	
H12	0.5015	−0.0518	0.1317	0.027 (3)*	
H13	0.2465	−0.0729	0.0435	0.027 (3)*	
C2	0.2905 (3)	0.3307 (2)	0.09280 (15)	0.0247 (3)	
H21	0.2217	0.2994	0.0114	0.033 (3)*	
H22	0.4786	0.3299	0.0972	0.033 (3)*	
H23	0.2305	0.4623	0.1149	0.033 (3)*	

### Atomic displacement parameters (Å^2^)

**Table:**

	*U*^11^	*U*^22^	*U*^33^	*U*^12^	*U*^13^	*U*^23^
S1	0.01277 (18)	0.02359 (19)	0.0170 (2)	−0.00180 (13)	0.00161 (12)	−0.00362 (15)
O1	0.0115 (5)	0.0353 (6)	0.0269 (6)	−0.0008 (4)	0.0030 (4)	−0.0069 (5)
C1	0.0159 (6)	0.0194 (7)	0.0209 (8)	0.0003 (5)	0.0030 (6)	0.0000 (6)
C2	0.0236 (7)	0.0198 (7)	0.0312 (9)	−0.0001 (6)	0.0042 (6)	0.0018 (6)

### Geometric parameters (Å, º)

**Table:**

S1—O1	1.5040 (10)	C1—H13	0.9800
S1—C1	1.7801 (14)	C2—H21	0.9800
S1—C2	1.7861 (15)	C2—H22	0.9800
C1—H11	0.9800	C2—H23	0.9800
C1—H12	0.9800		
			
O1—S1—C1	106.54 (6)	H12—C1—H13	109.5
O1—S1—C2	106.60 (7)	S1—C2—H21	109.5
C1—S1—C2	97.73 (7)	S1—C2—H22	109.5
S1—C1—H11	109.5	H21—C2—H22	109.5
S1—C1—H12	109.5	S1—C2—H23	109.5
H11—C1—H12	109.5	H21—C2—H23	109.5
S1—C1—H13	109.5	H22—C2—H23	109.5
H11—C1—H13	109.5		

### Hydrogen-bond geometry (Å, º)

**Table:**

*D*—H···*A*	*D*—H	H···*A*	*D*···*A*	*D*—H···*A*
C1—H11···O1^i^	0.98	2.42	3.3318 (18)	155
C1—H12···O1^ii^	0.98	2.42	3.3184 (17)	152
C1—H13···O1^iii^	0.98	2.47	3.4450 (19)	173
C2—H22···O1^ii^	0.98	2.61	3.4618 (18)	146

Symmetry codes: (i) −*x*, *y*−1/2, −*z*+1/2; (ii) *x*+1, *y*, *z*; (iii) −*x*, −*y*, −*z*.
